# “Functional outcomes and MRI-based tendon healing after (antero-) superior rotator cuff repair among patients under 50 years: retrospective analysis of traumatic versus non-traumatic rotator cuff tears”

**DOI:** 10.1186/s12891-023-06174-7

**Published:** 2023-01-21

**Authors:** Alexander Themessl, Timon Wagner, Marco-Christopher Rupp, Hannes Degenhardt, Klaus Woertler, Kate A. Hatter, Andreas B. Imhoff, Sebastian Siebenlist, Jonas Pogorzelski

**Affiliations:** 1 Department of Orthopaedic Sports Medicine, Technical University of Munich, Ismaninger Str. 22, 81675 Munich, Germany; 2 Department of Radiology, Technical University of Munich, Munich, Germany; 3 Department of Surgery/ Emergency Medicine, Duke University Hospital, Durham, NC USA

**Keywords:** Shoulder, Rotator cuff, Etiology, Trauma, Healing rate, Retear

## Abstract

**Background:**

Rotator cuff tears among patients under 50 years either result from an adequate trauma or are considered non-traumatic due to work-related or athletic overuse. The impact of these different mechanisms on postoperative functional outcomes and tendon healing has not yet been fully understood. Therefore, it was the purpose of this study to investigate the influence of etiology of (antero-)superior rotator cuff tears on postoperative outcomes and the healing rates after arthroscopic rotator cuff repair in a young patient population.

**Methods:**

Patients under 50 years who underwent arthroscopic rotator cuff repair between 2006–2017 for an anterosuperior rotator cuff tear with a minimum follow up of 24 months were included in this study. Revision surgeries or reconstructive concomitant procedures other than long head of the biceps tenodesis were excluded. Patients were divided into two groups according to the etiology of their rotator cuff tear (traumatic vs. non-traumatic). Demographic and outcome scores including the American Shoulder and Elbow Surgeons (ASES) score, the Constant Score (CS), bilateral strength measurements and postoperative tendon integrity evaluated on magnetic resonance imaging (MRI) were assessed and compared between both groups.

**Results:**

The mean follow up for this study was 55.6 months (24 – 158). Twenty-one patients (50.0%) had a traumatic RCT and 21 patients (50.0%) had a non-traumatic tear. Outcome scores did not differ significantly between groups. Strength measurements of the supraspinatus revealed significantly decreased force of the affected side as opposed to the contralateral side (*p* = 0.001), regardless of etiology. Retear rates were similar in both groups (37.5% and 33.3%, *p* = n.s.). Cuff integrity at follow-up was not predictive of superior scores or strength.

**Conclusion:**

Surgical treatment of traumatic and non-traumatic RCT yields good clinical results in patients under the age of 50. The etiology of the rotator cuff tear did not significantly affect postoperative outcomes or healing rates. About one third of the patients suffered from a retear postoperatively, however retears were not predictive of inferior outcomes at midterm follow-up.

**Study design:**

Level III.

**Trial registration:**

Retrospectively registered.

## Background

Rotator cuff tears are a frequent and potentially disabling pathology of the shoulder, not seldomly requiring surgical intervention [1–4]. While postoperative results are promising [5–7] both after open and arthroscopic reconstruction, patients’ outcomes can be affected by several risk factors, including increased preoperative tear size and fatty muscle infiltration, comorbidities and surgical repair techniques [1, 8–12]. Furthermore, patients’ age and the tear etiology (traumatic vs. non-traumatic) have been argued to affect postoperative tendon healing and outcome scores, however there remains uncertainty about the extent to which these two factors influence postoperative results [2, 13, 14].

Even though older age has been associated with increased tear and retear rates, potentially due to a changed biologic environment and thus inferior healing capacities [2, 13], postoperative subjective outcomes have been comparable to those reached by younger patients [3, 7, 15]. Furthermore, while traumatic tears theoretically have a superior healing potential and thus could yield better postoperative subjective outcomes compared to non-traumatic tears [14, 16], recent studies were unable to find such a difference with regard to the etiology of the rotator cuff tear [17–19]. Unfortunately, some heterogeneity regarding age and etiology in previous studies’ render the analysis of these aspects difficult. To better analyze and understand the effect of age and etiology, a separate evaluation is necessary. Thus, the primary purpose of this study was to assess the clinical outcomes and MRI-based healing rates among patients under the age of 50 years, and to detect potential differences with respect to the etiology of the initial rotator cuff tear. The secondary purpose was to correlate clinical outcomes to MRI findings (healed tendons vs. retear) at follow up. It was hypothesized that postoperative outcomes would ultimately differ with regard to the etiology of the RCT and tendon integrity at follow-up.

## Methods

This was a retrospective cohort study with ethical approval granted by the institution’s ethics committee (17/19 S-AS) and informed consent was obtained from all patients prior to participation. All patients younger than 50 years of age at the time of surgery who underwent rotator cuff repair for a symptomatic full thickness supraspinatus (SSP) or combined anterosuperior (supraspinatus and subscapularis) tear between 2006 and 2017 were included. Additionally, patients suffering from high grade partial thickness tears (Ellman [20] A/B 2 or 3) who underwent intraoperative completion and subsequent repair were also included. Surgery was indicated in the case of persisting or worsening of symptoms despite physical therapy during 3–6 months or upon patients´ explicit wish. The minimum postoperative follow up was 24 months. Patients with revision surgeries of the rotator cuff, concomitant pathologies (e.g. shoulder dislocations, fractures, stiff shoulders, calcified tendons), or additional reconstructive procedures (e.g. glenohumeral or acromioclavicular joint stabilization, arthrolysis) were excluded. Patients with preoperative signs of cuff tear arthropathy > 2 according to the Hamada classification [21] or glenohumeral osteoarthritis > 1 according to Samilson&Prieto [22] were also excluded from participation. Patients with a history of contralateral rotator cuff injury or reconstructive surgery were excluded from bilateral comparisons (e.g. force measurements).

For the purpose of this study and to test for our hypothesis, patients were divided into two different groups based on the etiology of the initial rotator cuff tear. Etiologies were grouped as either traumatic or non-traumatic. RCT were considered to be traumatic in case of an acute onset of symptoms after an adequate trauma (e.g. shearing of the tendons on the glenoid rim, when the maximal tolerated rotation angle is exceeded, passively forced external or internal rotation and abduction with a massive overstretching of anterocranial or posterocranial structures, axial compression and passive traction) to a previously asymptomatic and uninjured shoulder as described previously in the literature [14]. Correspondingly, rotator cuff tears without an adequate trauma or with a gradual onset of symptoms were considered non-traumatic, degenerative rotator cuff tears.

### Data collection

For each participating patient, demographic data (age, sex, affected side, arm dominance), medical and surgical information such as comorbidities, the affected tendon, surgical technique (single row vs. double row), concomitant injuries and procedures were gathered from hospital records. At final follow up, patient-reported outcome measures including the American Shoulder and Elbow Surgeons (ASES) score and the Constant Score (CS) were collected. A visual analog scale (VAS) was used to assess for pain. A standardized physical examination of the ipsilateral and contralateral shoulder was carried out by one of the authors (blinded for review). Examination included the assessment of passive and active glenohumeral range of motion using a goniometer and standard clinical testing for the rotator cuff. Force measurement of the affected muscles (supraspinatus and/or subscapularis muscle) was performed using a previously validated commercially available measuring device and calibration software (PCE-FB 5 K; PCE-Instruments GmbH, Germany) for analog-to-digital conversion and recording of the force data (sampling frequency, 40 Hz) [23, 24]. An Isobex isometric dynamometer (Cursor-AG, Switzerland) was used to assess supraspinatus strength and a custom-made force measuring plate was used for subscapularis muscle testing. Strength measurement (N) of the supraspinatus muscle was carried out according to the manufacturer’s instruction in a sitting position, with the arm positioned correspondingly to the Jobe test (90° abduction, 30° of flexion in the scapular plane with the hand in pronation). For isolated assessment of the subscapularis muscle, peak strength (N) of internal rotation was measured with the hand placed on the force measuring plate in the belly-press position.

Before supervised measurements, all patients were instructed on how to use the Isobex dynamometer and the force-measuring plate. Force measurements consisted of three consecutive measurements for the ipsilateral and contralateral side each. For final analysis, the mean values were calculated.

### Radiological Evaluation

In order to assess tendon integrity and fatty degeneration after rotator cuff repair, a high resolution 3-T magnetic resonance imaging (MRI) of the affected shoulder was performed at final follow up using a whole-body scanner (Ingenia, Philips Healthcare, Netherlands) for all patients. For radiologic evaluation, patients were in a supine position with their arm in neutral position. Imaging consisted of a standardized protocol including oblique sagittal, oblique coronal, and transverse planes in T1- and T2-weighted scans. All images were transferred on picture archiving communication system workstations (PACS, Easy Vision, Philips, Netherlands). Final MRI evaluation was carried out independently by two orthopedic surgeons (blinded for review).

Preoperative supraspinatus tears were classified according to the Patte classification [25] and subscapularis tears were classified according to the Fox & Romeo classification [26]. Postoperative tendon integrity was graded according to the Sugaya classification [27]. Tendons were judged as healed in cases of Sugaya types I-III. Tendons were considered as re-torn/not healed in cases of Sugaya types IV and V. Fatty degeneration of the affected muscles was assessed according to the Goutailler classification modified by Fuchs et al. [28]. For validity purposes, MR images were evaluated twice at an interval of 2 months and interrater/intrarater reliabilities were calculated. In the case of disagreements, grading was re-assessed in an additional session, and a joint decision was made for final evaluation.

### Operative technique and postoperative rehabilitation

All patients underwent surgery at a single institution between January 2006 and December 2017. Surgery was performed at this institution by one of the senior surgeons, who commonly have at least 10 years of experience in arthroscopic (shoulder) surgery. Each surgical technique was chosen with regard to the affected tendon, tear size, and tear configuration. In summary, surgery was performed under general anesthesia in the beach chair position. Initially, diagnostic arthroscopy was carried out via a posterior standard portal using a 30° arthroscope. Affected tendons, tear size, localization, and the degree of retraction were evaluated, and the glenohumeral joint was assessed for concomitant injuries. Prior to any reconstruction, debridement of the lesion was performed via additional anterolateral and posterolateral portals in order to create stable tear margins. If necessary, the tendon was released from surrounding tissue using an electrothermal or shaver device to achieve adequate tendon reduction and footprint coverage. For supraspinatus tears, the preferred method was a double row SpeedBridge technique (four 4.75-mm SwiveLock anchors, Arthrex, USA) to achieve best footprint fixation and protection from anchor failure. Margin convergence techniques were used in cases of L-shaped or crescent-shaped tear configurations, and a single row repair was used for small tears if it allowed sufficient footprint coverage. For cranial subscapularis repairs, one double loaded suture anchor (Biocorkscrew 5 mm, Arthrex, USA) was used. In larger tears, two anchors were applied in a single row technique [24, 29]. If a subacromial spur was present or in Acromion types 2 and 3 according to Bigliani et al. [30], subacromial decompression was performed using a shaver. If symptoms and arthroscopic findings suggested long head of the biceps tendon (LHB) pathology, tenotomy and, if desired by the patient, tenodesis (intraarticular or subpectoral) was performed.

Postoperatively, guided physical therapy was administered within the following limitations: the operated arm was supported with a 30° abduction pillow for 6 weeks. Limited passive range of motion was administered for six weeks, followed by consecutive progression to full active range of motion by the end of 9 weeks. In cases of additional subscapularis repairs, patients were instructed to avoid passive external rotation and active internal rotation and to restrict abduction to 90° in the scapular plane for 6 weeks, followed by a gradual progression to full active range of motion by the end of 9 weeks postoperatively. In cases of additional LHB tenodesis, no active elbow flexion was permitted within the first 6 weeks postoperatively.

### Statistical analysis

Statistical analysis for this study was performed using the SPSS software version 26.0 (IBM, statistics). Continuous variables are reported as mean ± standard deviation in case of normal distribution of the data, and for non-parametric variables, median and 1^st^—3^rd^ quartiles are presented. Categorial variables are reported as frequency (n) and percentage. Distribution of the variables was assessed using the Kolmogorov–Smirnov test and plot diagrams. For comparison of continuous variables between the study groups (two-tailed), the Mann–Whitney U test or an unpaired t-test were employed, while group comparison of categorical variables (two-tailed) was performed with the Chi-square test or the Fisher’s exact test, according to the data distribution. Cohen’s Kappa was used to assess for interrater and intrarater reliability [31]. Statistical significance was accepted when *p* < 0.05. A total sample size of *n* = 28 subjects to detect the minimal clinically important difference of the ASES score of 11.1 points [32] and a standard deviation of 10 points in order to achieve a statistical power of 0.8 was determined in an a priori power analysis, performed with G*Power (Erdfelder, Faul, Buchner, Lang, HHU Düsseldorf, Düsseldorf, Germany).

## Results

Between 2006 and 2017, 57 patients overall met the inclusion criteria for this study. Four patients were unable to schedule a follow up appointment and were excluded from analysis. Despite our best efforts, eleven patients could not be reached and were therefore considered lost to follow up. The remaining 42 patients (79%) were available for final follow up. The mean postoperative follow up was 55.6 months (range, 24–158). Twenty-one patients (50.0%) reported an adequate trauma prior to the onset of symptoms and twenty-one patients (50.0%) suffered a non-traumatic, degenerative rotator cuff tear. Baseline demographics and surgical characteristics were evenly distributed between both groups (Table 1).

**Table 1:** Baseline demographics and surgical information^*a*^

	Traumatic RCT (*n* = 21)	Non-traumatic RCT (*n* = 21)	*P*-value
Age, y (mean ± SD)	45.9 ± 3.6	43.7 ± 5.2	0.179
Sex			0.378
M	17 (81)	19 (90.5)	
F	4 (19)	2 (9.5)	
Dominant side			0.100
Yes	16 (76.2)	16 (76.1)	
No	5 (23.8)	5 (23.8)	
Tendon affected			0.355
SSP	9 (25)	12 (75)	
SSP + SSC	12 (75)	9 (25)	
Technique^*b*^			0.707
SR	5 (23.8)	4 (19.1)	
DR	16 (76.2)	17 (80.9)	
LHB procedure			0.100
Yes	20 (95.2)	20 (90.1)	
No	1 (4.8)	1 (9.9)	
ASD			0.707
Yes	16 (76.2)	17 (80.9)	
No	5 (23.8)	4 (19.1)	

^*a*^ Categorial data are presented as n (%). *RCT* Rotator cuff tear, *SSP* Supraspinatus, *SSC* Subscapularis, *SR* Single row, *DR* Double row, *LHB* Long head of the biceps tendon, *ASD* Arthroscopic subacromial decompression

^*b*^ Surgical technique for supraspinatus tendon repair

### Clinical outcomes

There was no statistically significant difference in VAS and PROMs (ASES, CS) between both groups (Table 2). Furthermore, tear characteristics (partial tear vs. full thickness tear; isolated supraspinatus tear vs. combined anterosuperior tear) did not significantly affect the CS or the ASES score.

**Table 2:** Postoperative outcome measures between patients with traumatic and non-traumatic RCT at follow up^*a*^

Outcome Scores		Traumatic RCT (*n* = 21)	Non-traumatic RCT (*n* = 21)	*P*-value
VAS		1.8 ± 1.1	1.6 ± 1.2	0.890
ASES Score		95 (74 -100)	97 (82–100)	0.779
Constant Score		80 (64 – 88)	82 (72 – 92)	0.434
Abduction		89 ± 9	91 ± 7	0.320
External rotation		58 ± 11	62 ± 11	0.328

^*a*^ Continous data are presented as mean ± standard deviation or median (1^st^ quartile – 3^rd^ quartile). Abduction in degrees, measured passively in the scapular plane, external rotation measured in degrees, passively at 0° of abduction. *RCT* Rotator cuff tear, *VAS* Visual analogue scale, *ASES* American shoulder and elbow surgeons, Abduction in the scapular plane

Overall, Passive abduction in the scapular plane was (mean ± SD) 90 ± 8 degrees and passive external rotation was 60 ± 11 degrees, with no between group differences detected (Table 2). All patients revealed statistically significant decreased abduction strength (66.0 ± 26.7 N vs. 81.2 ± 24.7 N; *p* = 0.001) on the operated shoulder compared to the contralateral shoulder. Patients with additional subscapularis repair (*n* = 21), had internal rotation strength which did not differ from the contralateral shoulder (80.7 ± 37.5 N vs. 80.6 ± 33.5 N).

### Radiological evaluation

Preoperative MRI was available for 40 patients (95.2%). There were four high-grade partial tears (19.0%) and 17 full thickness tears (81.0%) of the supraspinatus tendon among traumatic RCT, as opposed to twelve high-grade partial tears (57.1%) and nine full-thickness tears (43.9%) of the supraspinatus tendon among non-traumatic RCT (*p* = 0.011). In patients who underwent additional subscapularis repair during index surgery (*n* = 21), the majority of subscapularis tears were cranial tears (Table 3). Only two patients (5.0%) revealed a fatty degeneration grade 2 according to the Fuchs & Goutailler classification, and both of those patients had suffered a degenerative RCT. The remaining 38 patients (95.0%) showed either no signs of preoperative fatty degeneration (grade 0, 82.5%) or only some fatty streaks (grade 1, 12.5%).

**Table 3:** Preoperative tear tharacteristics^*a*^

	Traumatic RCT	Non-traumatic RCT	*P*-value
^*b*^Supraspinatus tears			0.470
Grade 1	13 (76.5)	5 (62.5)	
Grade 2	3 (17.7)	3 (37.5)	
Grade 3	1 (5.9)	0	
^*c*^Subscapularis tears			0.323
Grade 1	11 (84.6)	6 66.7)	
Grade 2	2 (15.4)	3 (33.3)	
Grade 3 & 4	0	0	

^a^ assessed on magnetic resonance imaging. Categorial data are presented as n (%). *RCT* Rotator cuff tear

^*b*^ tendon retraction of full thickness supraspinatus tears according to Patte [20]

^*c*^ subscapularis tear characteristic according to the Fox & Romeo classification [21]

At final follow up, a total of 33 patients (78.6%) were available for MRI evaluation. One patient had to be excluded from postoperative MRI evaluation due to claustrophobia, and one patient had to be excluded due to an acute shoulder injury prior to the follow up appointment. Postoperative MRI findings are listed in Table 4. Interrater and intrarater reliability for binary assessment (healed vs. not healed) of the affected rotator cuff reached a Cohen’s Kappa of 0.87 (CI 0.41—1.00) for interrater reliability and 0.86 (CI 0.67 – 1.00) for intrarater reliability. Overall, 20 tendons (64.5%) were judged as healed on follow up MRI (Sugaya I, II, III), whereas 11 tendons (35.5%) were judged as not healed (Sugaya IV, V). Retears only affected supraspinatus tendons. Individual patient characteristics (tear etiology, partial tear/full thickness tear, LHB procedures, additional ASD) were not associated with increased retear rates.

**Table 4:** Postoperative MRI findings^*a*^

Sugaya Classification	Traumatic RCT (*n* = 16)	Non-traumatic RCT (*n* = 15)	*P*-value
Healed	10 (62.5)	10 (66.7)	0.809
^*b*^Sugaya, n I:II:III	0:9:4	1:5:1	
Not Healed	6 (37.5)	5 (33.3)	
^*b*^Sugaya, n IV:V	4:2	2:3	

^*a*^ Categorial data are presented as n (%) unless otherwise noted. *RCT* Rotator cuff tear

^*b*^ n with regard to the distribution within each subcategory of the Sugaya classification [22]

Comparing patients with healed and retorn tendons at follow up, there was no statistically significant difference with regard to patient-reported outcome scores. Furthermore, force measurements revealed no inferior strength among patients with retorn tendons compared to patients with healed tendons (Table 5).

**Table 5:** Postoperative outcome measures between patients with and without healed tendons^*a*^

	Healed (*n* = 20)	Not healed (*n* = 11)	*P*-value
ASES score	96 (72 – 100)	93 (73 – 100)	0.867
Constant score	82 (64 – 89)	82 (63 – 90)	0.967
Abduction force, N	70.0 ± 29.5	59.1 ± 20.9	0.509

^*a*^ Continous data are presented as mean ± standard deviation or median (1^st^ quartile – 3^rd^ quartile). *ASES* American shoulder and elbow surgeons

## Discussion

The primary finding of this study was that in patients under the age of 50 years, the etiology of the rotator cuff tear did not affect postoperative outcomes and MRI-based healing rates after repair of (antero-)superior rotator cuff tears. The secondary finding was that tendon integrity on follow up MRI was not predictive of superior postoperative outcome scores or abduction strength compared to the uninjured side.

Among patients under the age of 50 years rotator cuff tears are uncommon and retears are rare, possibly due to a superior tissue quality and healing potential compared to older cohorts [11, 13]. In those patients the rotator cuff tear is either the direct result of a traumatic event or due to chronic tendon wear in the context of heavy labor or athletic overuse [5, 16–18, 33–35]. But despite a number of studies having previously addressed this aspect, debate remains whether or not etiology plays a role for postoperative patient outcomes and healing rates. In a previous study comparing both etiologies of RCT, Braune et al. [16] found superior postoperative results in the Constant score among patients with a traumatic tear as opposed to patients with non-traumatic tears (94.1 vs. 75.3). However, owing to their definition of traumatic tears (patient age < 50 years), those patients were substantially younger than the patients with degenerative tears in that study (mean 34.2 years vs. 54.1 years). Therefore, age could not be neglected as a potential confounder for the differences in scores. Other studies compared postoperative outcomes between traumatic and non-traumatic RCT in patients of a similar age without finding significant group differences. For example, Kukkonen et al. [19] and Tan et al. [18] reported similar clinical results on the Constant score, range of motion, and during strength testing among slightly older patients with a mean age between 57—60 years. Lin et al. [17] reported equivalent subjective outcome scores (ASES, CS, Simple Shoulder Test) between patients.

(< 45 years) with and without a traumatic event leading to their injury, however without assessing for radiological tendon-integrity. In the present study and similar to the previous studies, we were not able to detect significant differences in patient-reported outcome scores (ASES score, CS), nor did we find differences during strength measurements between traumatic and non-traumatic rotator cuff tears, thus rejecting our alternative hypothesis. However, whether or not etiology may relevantly influence postoperative outcomes may in fact be dependent on the timing of surgery and patient age. While there is controversy with regard to the best timing for surgical repair, evidence suggests that an early treatment is beneficial, particularly in traumatic tears [8, 36, 37]. Early surgical repair is intended to prevent tendon retraction, muscle atrophy, and fatty degeneration. However, acute traumatic injuries are accompanied by an inflammatory reaction, possibly augmenting postoperative tendon healing in the early posttraumatic phase [14]. Immediate surgical treatment is not always wished for or possible to perform, the potentially advantageous acute posttraumatic interval may often be missed [8, 10, 18, 38]. In the current study only three patients with traumatic tears presented within the first 6 weeks of their initial injury and therefore the potential benefits of acute tendon repair would not be expected. Furthermore, the influence of both traumatic and non-traumatic RCT may vary with respect to patient age. Increasing age is generally associated with a variety of biomechanical features such as osteoporotic bone, inferior tendon composition, and diminished vascular supply, which may adversely affect postoperative tendon healing and patient outcomes regardless of the etiology of the rotator cuff tear [39, 40]. But at what age these processes set in and begin to adversely affect surgical outcomes is unknown. Therefore, while patients in their fourth and fifth decades may not be biologically young nor old, even traumatically torn tendons may have already undergone (age-dependent) tendon wear which might compromise surgical results [17–19]. This concept of acute-on-chronic lesions is not new, but its potentially confounding implication must be accounted for when evaluating outcomes of traumatic RCT at a certain age [19]. In summary, potential biological benefits of traumatically torn tendons may in fact be lost due to a delay of treatment and with increasing patient age. Both aspects could explain the similar outcomes between patients with traumatic and non-traumatic tears.

In the present study, a retear of the previously reconstructed supraspinatus tendon was detected in one third of the patients at follow up. Similar rates are reported throughout the literature, ranging from 12% to well over 50%, depending on length of follow up, initial tear size, surgical technique, and age [18, 41–46]. While recent systematic reviews generally suggest that retears of the rotator cuff are associated with inferior postoperative outcomes, it remains unclear why some of the patients with a retear become symptomatic and potentially require revision surgery while others remain clinically inapparent, maintaining fairly satisfying outcomes in certain studies [11, 23, 43, 47–55]. In the current study, retears were not associated with inferior ASES or Constant scores compared to patients with healed tendons. The patients with a retear showed a tendency towards decreased strength of the affected shoulder compared to the contralateral shoulder, but without reaching statistical significance. It remains unclear why a tendon retear did not affect postoperative outcomes. Increasing age and need for workers’ compensation have been shown to be negative predictive factors after a retear of the reconstructed rotator cuff [53]. While none of the patients in our study received workers’ compensation with regard to their shoulder injury, it has to be considered that even though younger patients may place higher demands on their postoperative functional results than older patients, their intact force couple and deltoid muscle may also be more capable of temporary compensation for a torn supraspinatus muscle. What is more, all patients, regardless of tendon healing, showed significantly inferior abduction strength compared to the uninjured arm at follow up. Therefore, it is possible that a retorn tendon might not have sufficed to sufficiently diminish strength in order to reach statistical significance at this mid-term follow-up.

Although this study presents interesting findings, it is not without limitations. This was a retrospective study and despite our best efforts, patients were lost to follow up, hence carrying the risk of selection bias. Additionally, the patient cohort was heterogenous with a large proportion of anterosuperior RCT and a relevant amount of concomitant LHB procedures. As these procedures were divided up equally between both groups we do not consider this to have confounded the results. Furthermore, patients who reported traumatic injuries might have in fact suffered from acute on chronic injuries despite the presence of an adequate trauma and an acute onset of symptoms, and immediate treatment of traumatic tears was seldomly achieved thus potentially confounding the outcomes of these patients. Longer follow up might have been needed to detect significant differences between healed and not healed rotator cuffs as mechanisms to compensate for a torn tendon might still have been sufficient at this mid-term follow-up. Lastly, future studies might take into consideration the effects of a more immediate treatment particularly in traumatic RCT.

## Conclusion

The etiology of the rotator cuff tear did not significantly affect postoperative outcomes and healing rates in a relatively young cohort. About one third of the patients revealed a full thickness retear, but no significant association could be established between structural integrity of the rotator cuff and clinical outcomes at mid-term follow up.

## Data Availability

All data generated or analysed during this study are included in this published article or are available from the corresponding author on reasonable request.

## Abbreviations

ASES: American shoulder and elbow surgeons
CS: Constant score
MRI: Magnetic resonance imaging
N: Newton
N.S: Not significant
RCT: Rotator cuff tear
SSC: Subscapularis tendon
SSP: Supraspinatus tendon
VAS: Visual analog scale
